# Severe Hypercalcemia Caused by Calcium-Alkali Syndrome after 15 Years of Postoperative Hypoparathyroidism in a Patient with Undiagnosed Hyperaldosteronism

**DOI:** 10.1155/2024/3067354

**Published:** 2024-02-16

**Authors:** Natália Diel Boufleuer, Dimitris V. Rados, Tatiana Zambonato, Clara K. Maraschin, Beatriz D. Schaan

**Affiliations:** ^1^Hospital de Clínicas de Porto Alegre, Rua Ramiro Barcelos 2400, Zip Code 90035-903, Porto Alegre, RS, Brazil; ^2^Internal Medicine Division, Hospital de Clínicas de Porto Alegre, Rua Ramiro Barcelos 2400, Zip Code 90035-903, Porto Alegre, RS, Brazil; ^3^Post-Graduate Program in Medical Sciences: Endocrinology, Universidade Federal do Rio Grande do Sul, Rua Ramiro Barcelos 2400, Zip Code 90035-903, Porto Alegre, RS, Brazil; ^4^Hospital Moinhos de Vento, Porto Alegre, RS, Brazil; ^5^Universidade Federal do Rio Grande do Sul, Rua Ramiro Barcelos 2400, Zip Code 90035-903, Porto Alegre, RS, Brazil; ^6^Endocrine Division, Hospital de Clínicas de Porto Alegre, Rua Ramiro Barcelos 2400, Zip Code 90035-903, Porto Alegre, RS, Brazil; ^7^National Institute of Science and Technology for Health Technology Assessment (IATS)-CNPq/Brazil, Rua Ramiro Barcelos 2350, Zip Code 90035-903, Porto Alegre, RS, Brazil

## Abstract

**Introduction:**

The triad of hypercalcemia, metabolic alkalosis, and acute kidney injury associated with ingesting high doses of calcium and absorbable bases characterizes the calcium-alkali syndrome. *Clinical Case*. We report the case of a patient with postthyroidectomy hypoparathyroidism 15 years ago due to differentiated thyroid cancer who presented with severe hypercalcemia. He had adequate control of calcemia for many years on treatment with calcitriol and calcium carbonate and hypertension treated with amlodipine, losartan, and hydrochlorothiazide. After a period of loss to follow-up, he suddenly presents with severe hypercalcemia, metabolic alkalosis, and loss of renal function. Upon hydration and withdrawal of calcitriol and calcium replacements, hypercalcemia resolved. The etiological investigation identified no granulomatous or neoplastic diseases, but an aldosterone-producing adrenal incidentaloma was found. The cause of hypercalcemia in this patient was calcium-alkali syndrome due to calcium carbonate replacement potentiated by hydrochlorothiazide and primary aldosteronism. Six months after the hospitalization and suspension of calcium and vitamin D, the patient returned to hypocalcemia, reinforcing the diagnosis.

**Conclusion:**

Although seldom described, the calcium-alkali syndrome is an expected complication for individuals with postoperative hypoparathyroidism, as they require lifelong calcium and vitamin D supplementation. This case also shows the importance of hydrochlorothiazide use and primary aldosteronism as possible triggers of life-threatening hypercalcemia.

## 1. Introduction

Hypercalcemia can be a diagnostic challenge for the physician, leading to extensive, uncomfortable, and costly patient evaluations. The list of potential etiologic causes is extensive; it includes straightforward diagnoses, such as primary hyperparathyroidism, and severe conditions, such as multiple myeloma, disseminated granulomatous diseases, and occult neoplasms [1]. In some situations, the patient does not appear to fit into traditional diagnostic algorithms, and extensive investigation is needed [2].

Among the causes of hypercalcemia, the calcium-alkali syndrome is sometimes overlooked but is particularly important in some scenarios. Historically, this syndrome was called “milk-alkali” because gastric ulcer treatment with a combination of milk, magnesium carbonate, and sodium bicarbonate was the main trigger [3]. The current definition involves the triad of hypercalcemia, metabolic alkalosis, and acute kidney injury related to ingesting high doses of calcium and absorbable bases. The main clinical context for its diagnosis is dietary calcium carbonate replacement or supplementation, especially in postmenopausal women. However, it can also occur while being treated for hypoparathyroidism, mainly when high doses of calcium carbonate are associated with loss of renal function. In addition, any situation with excessive calcium ingestion combined with an alkaline element may promote calcium-alkali syndrome development [4].

Patients with hypoparathyroidism who develop hypercalcemia represent a unique diagnostic challenge. At first sight, there is a paradox in this situation: the excessive calcium blood level is unexpected in patients with hypoparathyroidism. As parathyroid hormone (PTH) is absent, there is reduced mobilization of calcium from bone stores and renal resorption, with a tendency to hypocalcemia [5]. However, in many situations, hypercalcemia has other mechanisms that are independent of the PTH effect.

This report presents a case of hypercalcemia in a patient with permanent hypoparathyroidism caused by calcium-alkali syndrome, where several associated contributing factors were identified. Based on it, we explored and discussed the literature on the subject and made recommendations regarding managing patients predisposed to this condition.

## 2. Case Report

A 47-year-old male university professor, previously hypertensive with adequate blood pressure control, developed hypoparathyroidism after a total thyroidectomy for papillary thyroid carcinoma performed 15 years ago. More than one year after the procedure, he had a PTH of less than 2.5 pg/mL and calcium of 7.9 mg/dL (Table 1). At the time, he was asymptomatic, with no changes in the physical examination suggestive of hypocalcemia, and was using levothyroxine 150 mcg, calcium carbonate 1000 mg/day, and calcitriol 0.25 mcg, in addition to antihypertensive drugs (amlodipine, losartan, and hydrochlorothiazide). Medication adjustments led to calcium-level normalization. About ten years after starting follow-up, the patient had a total calcium of 9 mg/dL (Table 1), using calcium carbonate 1500 mg/day and calcitriol 0.25 mcg. He remained asymptomatic without clinical, imaging, or laboratory signs of thyroid carcinoma recurrence.

**Figure 1:**
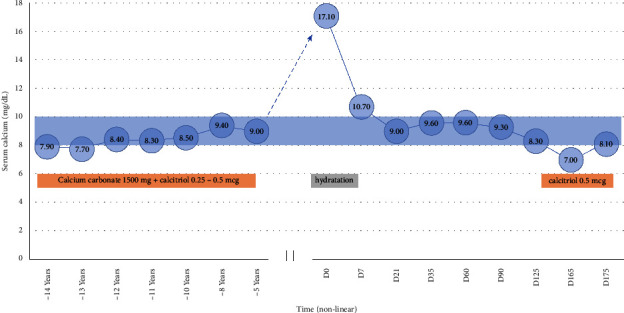
Patient's calcium over time and treatments performed. The boxes show the treatment over time. The blue area represents calcium reference range. D: day; time data: D0: hypercalcemia diagnosis; (−): time before D0.

After a few years with irregular medical follow-up and no calcium-level evaluation, the patient returned for a consultation with a cardiologist with vague complaints of mental confusion, fatigue, and weight loss (approximately 3 kg). Laboratory tests performed at that time showed worsening of renal function (creatinine 7.31 mg/dL) and hypercalcemia (calcium 17.1 mg/dL) (Figure 1). The patient was hospitalized for management of hypercalcemia, receiving vigorous intravenous hydration, and having suspended calcium carbonate and calcitriol. During hospitalization, alternative causes for loss of renal function were evaluated, ruling out primary or secondary glomerular damage; there was also no evidence of direct tubular damage. The finding of bicarbonate (alkaline reserve) at the upper limit of normal was noteworthy despite the loss of renal function. The patient evolved with progressively lower calcium levels and was discharged with a calcium of 10.7 mg/dL (Figure 1) and creatinine of 5.9 mg/dL after four days of hospitalization, with an indication for follow-up investigation on the etiology of hypercalcemia on an outpatient basis.

The etiologic investigation included serum protein electrophoresis and urine light-chain investigation, which ruled out paraproteinemia as a cause of hypercalcemia. In addition, imaging excluded solid neoplasms: (a) chest tomography showed no tumescent lesions; (b) abdomen tomography identified nephrolithiasis on the left kidney and a nodular image in the left adrenal measuring 1.2 cm of indeterminate nature; (c) upper digestive endoscopy had no alteration, and (d) colonoscopy identified a tubular adenoma with low-grade dysplasia.

Bone scintigraphy and whole-body PET-CT were also performed, with no image suggestive of neoplasia. The dosage of PTH-related protein (PTHrp) was less than 1.02 pg/mL.

The adrenal nodule was associated with excessive aldosterone production, reduced renin secretion, and elevated aldosterone/plasma renin activity ratio (Table 1). It was decided not to perform a suppression test because the patient still had compromised renal function, low volume tolerance, and adequate blood pressure control with current medications.

After 30 days of the acute condition, the patient remained with controlled calcemia (Figure 1) without additional intervention. The diagnosis of the calcium-alkali syndrome was based on loss of renal function, alkalosis even with a severe reduction in glomerular filtration rate (Table 1), unmonitored use of calcium carbonate and vitamin D in the setting of diuretic usage, and investigations ruling out other secondary causes of hypercalcemia. At last, the favorable evolution of hypercalcemia also reinforces this diagnosis.

Hydrochlorothiazide usage and primary hyperaldosteronism are events peculiar to this case and contributors to alkalosis and hypercalcemia. Finally, six months after admission for hypercalcemia, the patient had symptomatic hypocalcemia (positive Trousseau maneuver), reinforcing the diagnosis. In this context, calcitriol 0.5 mcg was restarted to resolve symptoms and control calcemia without additional calcium carbonate supplementation.

## 3. Discussion

We present a case of PTH-independent hypercalcemia after a long period of hypoparathyroidism with adequate control with calcium carbonate, calcitriol, and hydrochlorothiazide. Initial tests did not show increased vitamin D metabolites, ruling out vitamin D intoxication. While waiting for the PTHrp result, numerous tests excluded neoplasms and other secondary causes of hypercalcemia. With low PTHrp, it was possible to rule out the hypothesis of malignancy-related hypercalcemia, mainly because there was no evidence of a malignant cause, considering the extensive investigation. Moreover, no recovery of parathyroid function was observed. Forasmuch as the favorable evolution and loss of renal function, metabolic alkalosis, and previous calcium carbonate and vitamin D use, we concluded that this is a calcium-alkali syndrome.

Hypercalcemia is a common problem in clinical practice, and its etiology is seldom challenging. More than 90% of hypercalcemia cases are attributable to primary hyperparathyroidism and malignancies [6]. The first step in its etiological investigation is to assess PTH levels to define whether it is PTH-dependent (e.g., primary hyperparathyroidism) or PTH-independent hypercalcemia (e.g., malignant disease, vitamin D intoxication, and granulomatous diseases). In the presence of low PTH levels, PTHrp and vitamin D metabolites (1,25-OH-vitamin D and 25-OH-vitamin D) provide further insights into the mechanism of hypercalcemia. At last, if, after these tests, the etiology remains elusive, other rarer causes of hypercalcemia should be investigated [7].

The triad of hypercalcemia, metabolic alkalosis, and acute kidney injury associated with ingesting high doses of calcium and absorbable bases defines the calcium-alkali syndrome. Individuals with advanced age or chronic kidney disease are at greater risk of developing this syndrome. They are more susceptible to volume depletion and reductions of glomerular filtration rate [8, 9]. Calcium carbonate contributes to the onset of calcium-alkali syndrome in two ways: first, it is a source of exogenous calcium, and, in the presence of contributing factors, it can trigger hypercalcemia. Second, calcium carbonate increases bicarbonate levels (reducing renal excretion) and contributes to metabolic alkalosis [4]. Once established, hypercalcemia and metabolic alkalosis interact in a sequence that maintains elevated calcemia. Hypercalcemia promotes total body water depletion, reduces the glomerular filtration rate, and increases sodium excretion; this leads to increased bicarbonate reabsorption and metabolic alkalosis. Alkalosis increases calcium reabsorption in the distal nephron, further aggravating hypercalcemia [8]. The entire sequence described above is worsened by hypoparathyroidism, as the lack of PTH hinders renal calcium excretion [10].

Thiazide diuretics block the sodium-chloride cotransporter in the apical membrane of the distal convoluted tubule, promote calcium reabsorption, and contribute to alkali overload. In addition, these drugs decrease the glomerular filtration rate and promote the maintenance of alkalosis by stimulating bicarbonate reabsorption in the proximal renal tubule. Thus, hypercalcemia originating in the setting of chronic calcium overload becomes more apparent with the use of thiazides [4]. Primary aldosteronism may act similarly, exacerbating alkalosis and, consequently, hypercalcemia.

In addition to this possible causative role, aldosterone and hypercalcemia seem to have a bidirectional relation. Previous data indicate that hypercalcemia can stimulate aldosterone secretion [11, 12]. As such, in this patient, the high aldosterone levels may be in fact a manifestation of hypercalcemia that would otherwise be undetected without the latter. However, this effect seems to be mediated, at least in part, through PTH, which is absent in this case [12]. Also, aldosterone can induce urinary calcium excretion, an effect that would be protective by reducing serum calcium levels. Irrespective of the direction of the effect, the present unusual case brings up this calcium metabolism/aldosterone secretion relationship. Moreover, it is suggested that, at least in part, this is a PTH-independent effect. The state of hyperaldosteronism may have contributed to the development of the calcium-alkali syndrome through metabolic alkalosis; however, this relation may be bidirectional, and the small magnitude of alkalosis suggests that this was a minor effect.

Patients with permanent postoperative hypoparathyroidism require lifelong calcium and vitamin D supplementation to prevent symptomatic hypocalcemia. The goal is to reach the lower limit of normal calcemia while avoiding hypercalciuria. The associated use of thiazide diuretics is common to maintain normal serum calcium levels and avoid chronic hypercalciuria [4]. Its use in the present case was also part of the hypertension treatment, a condition with high prevalence.

This case stands out due to the combination of factors for developing the calcium-alkali syndrome that occurred in a successive and additive way. For 15 years, the patient kept hypoparathyroidism controlled with calcium carbonate and calcitriol replacement. His elevated blood pressure was treated with an angiotensin receptor blocker and a thiazide diuretic, which are the mainstay treatment of hypertension but are associated with an increased risk of calcium-alkali syndrome. Early-stage chronic kidney disease (stage G3) was first noticed one year before the acute event. After several years of regular medical follow-up with annual calcium dosages, the patient did not undergo control exams for five years. Calcium levels were probably already rising, but the lack of regular endocrinological follow-up did not allow for early identification. In addition to all these factors, the patient also had previously unknown primary aldosteronism, discovered incidentally through a computerized tomography scan of the abdomen showing an adrenal nodule. The confirmatory test was deferred due to safety, patient preferences, and the high probability of being an aldosterone-producing adenoma [13]. The state of hyperaldosteronism probably contributed to the development of the calcium-alkali syndrome through metabolic alkalosis.

## 4. Conclusion

Individuals with postoperative permanent hypoparathyroidism, a frequent complication of total thyroidectomy, require calcium and vitamin D supplementation (sometimes in high doses) to prevent hypocalcemia; paradoxically, this treatment can promote hypercalcemia. The calcium-alkali syndrome can occur at any time during the treatment of hypoparathyroidism [14]. Importantly, this report shows that two situations expected in some patients with hypertension (hydrochlorothiazide usage and primary aldosteronism) may trigger life-threatening hypercalcemia. Therefore, patients undergoing calcium and vitamin D replacement should regularly monitor serum calcium levels, renal function, and acid-base balance. This follow-up may detect subtle hypercalcemia and prevent the complete calcium-alkali syndrome along with possibly life-threatening loss of renal function from emerging.

## Figures and Tables

**Table 1:** Laboratory tests presented chronologically.

	Reference range	−14 years	−13 years	−12 years	−11 years	−10 years	−8 years	−5 years	−1 years	D0	D7	D21	D35	D60	D90	D125	D165	D175
Hemoglobin (g/dL)	13.2–18.0	16.5	15.8			15.9		16	14.7	10.2	8.9			10	11.1	11.6		11.8
Hematocrit (%)	39.1–50	48	46.2			45.5		46.1	42	30.1	26.9			29.9	33.2	35.5		35.8
MCV (fL)	80–99	85	85.3			85		85.8	84.2	88.5	89.4			89.9	87.1	87		86.9
Leucocytes (*μ*L)	3700–9500	6500	5670			6010		6310	6000	8740	8120			5340	6570	6230		7570
Platelets (*μ*L)	140000–400000					220000		211000	223000	294000	299000			244000	240000	223000		240000
Creatinine (mg/dL)	0.66–1.25			0.94		1.01		1.12	1.48	7.31	5.9	4.3	3.8	3.6	3	3		2.3
eGFR (ml/min/1.73 m^2^)	>60			89.7		77.9		68	49	7.6	9.8	15	16.3	17.3	21.4	21.4		29
Potassium (mmol/L)	3.5–5.1							4.1	3.7	4.2	4.2		4.4	3.9	3.7	4.4		4.2
Sodium (mmol/L)	137–145									135	140		142	140	141	142		139
Calcium (mg/dL)	8.4–10.2	7.9	7.7	8.4	8.3	8.5	9.4	9		17.1	10.7	9	9.6	9.6	9.3	8.3	7	8.1
Albumin (g/dL)	3.5–5.0		4.43							4.4	4			4.3	4.3			
Bicarbonate (mmol/L)	22–30									33	28		25	27		28		26
Phosphate (mg/dL)	2.5–4.5	5.8	5.18	4.8	4.73	4	3.9	3.8		4.6	3.8	4.7	5	4.8	4.7	4.3	5.6	4.6
Glycemia (mg/dL)	70–100	89	93		94	95		94	97	95					96	78		
TSH (mUI/L)	0.51–4.30	1.99	0.03		<0.01	<0.05	0.72	1.7	1.6	0.16				2.35			2.01	
Free T4 (ng/dL)	0.93–1.7	1.28	1.5		2.1	1.6	1.5	1.4	1.4	1.3				1.19			1.18	
Thyroglobulin (ng/ml)	1.1–55	0.39		<0.2			0.2											
PTH (pg/mL)	4.0–65.0	<2.5		6	9					2.9	5.3			5.8		9.9	11.9	11
25-OH-VitD (ng/mL)	20–60									39	36.6			34		48.6		
1.25-OH-VitD (pg/mL)	19.9–79.3										10			<15				
Aldosterone (ng/dL)	2.5–39.2												64.1			76.9		
Renin (ng/mL/H)	0.04–4.95												1			0.8		
Urinary calcium (mg/24 h)	100–300			269			364			423		156		229		79		

D: day; eGFR: estimated glomerular filtration rate; MCV: mean corpuscular volume; T4: thyroxine; PTH: parathyroid hormone; TSH: thyroid-stimulating hormone; VitD: vitamin D. Time data: D0: hypercalcemia diagnosis; (-): time before D0.

## Data Availability

All data were included in the article.
